# Influence of Noise in Computer-Vision-Based Measurements on Parameter Identification in Structural Dynamics

**DOI:** 10.3390/s23010291

**Published:** 2022-12-27

**Authors:** Mariusz Ostrowski, Bartlomiej Blachowski, Grzegorz Mikułowski, Łukasz Jankowski

**Affiliations:** Institute of Fundamental Technological Research, Polish Academy of Sciences, 02-106 Warsaw, Poland

**Keywords:** computer vision, smartphone camera, system identification, model updating, uncertain bolted connections

## Abstract

Nowadays, consumer electronics offer computer-vision-based (CV) measurements of dynamic displacements with some trade-offs between sampling frequency, resolution and low cost of the device. This study considers a consumer-grade smartphone camera based on complementary metal-oxide semiconductor (CMOS) technology and investigates the influence of its hardware limitations on the estimation of dynamic displacements, modal parameters and stiffness parameters of bolted connections in a laboratory structure. An algorithm that maximizes the zero-normalized cross-correlation function is employed to extract the dynamic displacements. The modal parameters are identified with the stochastic subspace identification method. The stiffness parameters are identified using a model-updating technique based on modal sensitivities. The results are compared with the corresponding data obtained with accelerometers and a laser distance sensor. The CV measurement allows lower-order vibration modes to be identified with a systematic (bias) error that is nearly proportional to the vibration frequency: from 2% for the first mode (9.4 Hz) to 10% for the third mode (71.4 Hz). However, the measurement errors introduced by the smartphone camera have a significantly lower influence on the values of the identified stiffness parameters than the numbers of modes and parameters taken into account. This is due to the bias–variance trade-off. The results show that consumer-grade electronics can be used as a low-cost and easy-to-use measurement tool if lower-order modes are required.

## 1. Introduction

Information about structural stiffness, which is crucial in structural health monitoring (SHM), is better reflected by dynamic displacements than by accelerations, as the latter are strongly affected by other parameters, e.g., mass [1]. Nowadays, simple cameras mounted on stands or on unmanned aerial vehicles (UAVs) have potential in both monitoring and inspection of structural conditions [2,3]. The application range of computer vision in infrastructure assessment is very wide, and this paper focuses on estimation of dynamic displacements for parametric identification of structures.

Feng and Feng indicated several advantages of CV displacement measurement over techniques employing traditional sensors [4]:

- To be installed, a camera does not require physical access to the structure. Hardly accessible structures can be observed remotely using zoom lenses [5,6]. In comparison to contact sensors, this significantly reduces the monitoring costs of many critical parts of structures such as cables, as indicated in [7].
- Compared to GPS technology, which also does not require a physical reference point near the monitored structure, CV measurements are much more accurate [8].
- As opposed to traditional point-wise sensors, a camera is able to simultaneously capture the motion of multiple points [9]. Moreover, it is possible to select these points in the recorded video after the measurement session.

Apart from the advantages of CV measurements, some weaknesses of this technique should also be noted. Measured higher-frequency components of motion often become highly contaminated by noise due to equipment limitations such as insufficient camera resolution or a relatively low sampling frequency [10]. Thus, sensing dynamic displacement using a camera requires an appropriate video-processing algorithm to reduce the influence of these limitations.

**Figure 1:**
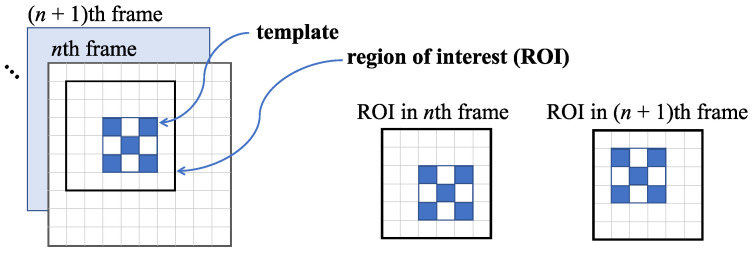
Scheme of a template tracked within the selected ROI in subsequent video frames.

Template matching methods are the most popular displacement estimation techniques due to the possibility of achieving subpixel-level accuracy. Generally, in template matching, the tracked object is represented by a preselected image region, namely, a template, of a selected frame of the video (usually the first one). The displacement of this template is determined in the subsequent video frames by searching for and matching this template with the most similar region within a search area in the current video frame, called the region of interest (ROI). The template matching process is illustrated in Figure 1.

Based on the matching technique, template matching methods can be classified into area-based and feature-based template matching [11]. In area-based template matching, both the template and the ROI are represented by their pixel intensities. Matching of the template within the ROI is performed by maximization of the cross-correlation function or by minimization of the error function, e.g., sum of squared differences [12]. In feature-based template matching, both the template and the ROI are usually represented by a set of characteristic points, called the keypoints. They are matched according to the information about their vicinity encoded in descriptors, such as the fast retina keypoint (FREAK) [13]. Area-based template matching usually provides more accurate displacement estimation if good illumination is provided, whereas feature-based template matching is more robust with respect to changes in illumination, scale, rotation, etc. [14]. It can thus be more suitable for outdoor field measurements.

Errors caused by the limited resolution of the camera can be reduced by using subpixel techniques [15]. Feng et al. showed that the displacement estimation error is unacceptable when a template is matched with the accuracy of the pixel size in a video [16]. They demonstrated that with their proposed upsampling technique, the quantization error can be reduced with a simultaneous decrease in the subpixel size. In practice, it is possible to achieve a precision of displacement estimation even near to 0.01 pixels [17] with methods involving a subpixel precision search. In [18], the fundamental natural frequency of the monitored structure was identified even from vibrations of 0.21 mm amplitude from a distance of over 175 m, which amounts to a precision of 1/175 pixel. Interpolation of the cross-correlation function in the vicinity of its maximum provides accurate results, but it must be guaranteed that the interpolation function has the maximum [19].

Camera calibration is required to transform the displacements in the video (expressed in pixels) into physically meaningful information. In the simplest case, if the direction of the camera view is perpendicular to the filmed surface, then only a scaling factor is required. This can be determined from the known physical dimensions of the filmed structure and its pixel size in the video frames [20]. When the camera axis cannot be positioned perpendicularly to the filmed object, then the tilt angle needs to be included in calculations, e.g., as proposed by Pen et al., where a laser rangefinder is additionally used in the camera calibration procedure [21]. If three dimensional (3D) displacement is to be measured, then a more sophisticated camera calibration procedure can be required. Park et al. proposed a methodology for the calculation of 3D displacements from two dimensional displacements obtained with multiple cameras [22]. The methodology requires a “T”- or “L”-shaped wand with attached markers to be placed in the field of view near the monitored structure in order to calibrate the cameras. Narazaki et al. proposed a model-informed approach for 3D displacement estimation with the use of a single camera. Instead of the wand or a calibration panel, it employs the known dimension of the structure associated with the selected points in the video frame [9].

Another important issue in CV measurement is the camera motion caused by ground vibration. This problem is not always significant, as shown by Feng and Feng, where the error resulting from camera motion was negligible [23]. However, camera motion can, in general, significantly decrease the measurement accuracy. Usually, reference targets that do not belong to the measured object are employed to estimate the camera motion, e.g., as shown by Yoneyama and Ueda [24]. More than one reference target allows estimation of both translation and rotation of the camera, and thus more effectively reduces the related errors [25]. Another kind of camera motion is present when the camera is mounted on a UAV, as shown in [26]. In this work, a methodology based on the Fourier spectrum of the relative displacement of two adjacent points on the cable is proposed. This allows the influence of the UAV motion to be effectively reduced.

There are also other sources of errors. In remote measurements from a significant distance, heat haze can distort the refractive index of the air and affect the measurement accuracy [27]. The widely used complementary metal-oxide semiconductor (CMOS) sensors do not register the whole image at once, as opposed to charge-coupled device (CCD) sensors, but they usually scan the image row-by-row or column-by-column. This can result in the rolling shutter effect if relatively high-speed motion is recorded. Lee proposed a real-time algorithm for video stabilization and compensation of the rolling shutter effect to be used with low-cost consumer cameras [28]. If the monitored structure vibrates with a frequency higher than half of the camera sampling frequency, temporal aliasing occurs. This can often be noticed with consumer cameras, which usually have a lower sampling frequency than professional high-speed cameras. Moreover, consumer cameras can have an inaccurate sampling frequency, i.e., different from the one declared in the camera specifications. These two issues appeared in a low-cost CV identification system proposed by Yoon et al., as discussed in [29]. The small focal length common in consumer-grade smartphone cameras causes bigger displacement estimation errors for a fixed distance [16]. However, a smaller focal length also extends the field of view of the camera; thus, the camera can be moved closer to the recorded object while still registering all the measurement points [30]. This allows a more profitable trade-off between the resolution and the field of view. The lower pixel size of low-cost cameras makes them more sensitive to changes to illumination in field measurements.

Due to the mentioned advantages of low-cost cameras, especially smartphone cameras, they are frequently used for estimation of displacements and identification of structural dynamic properties [31,32,33,34]. Min et al. showed that CV measurements with a smartphone camera can have accuracy comparable to that of a laser displacement sensor (LDS) [31]. Li et al. indicated that a smartphone camera allows interstory drift of buildings to be accurately measured during an earthquake [33]. The results obtained with the tested smartphones were comparable with those of an LDS, too. Zhu et al. compared the natural frequencies identified with the aid of a smartphone to those obtained using an accelerometer and simulations [34].

The dynamic displacement estimation techniques discussed above are often used for identification of modal parameters of structures, which can subsequently be used for model updating. Among the variety of methods for experimental modal analysis, stochastic subspace identification using time-domain data (SSI-DATA) is widely recognised as accurate and robust [35]. It is devoted to system identification when excitations are unknown; hence, it is suitable for CV measurements.

Experimentally identified modal parameters are often used in the model updating procedure. Model updating generally has one of two aims: (1) calibration of the model, which aims to provide accurate reproduction of the output of the real system, or (2) identification of unknown structural parameters [36].

It is known that the results of a model updating procedure are affected not only by the measurement noise and modelling errors but also by ill-conditioning of the problem, and thus numerical regularisation of the solution (or imposing additional constraints) enhances the quality of the solution. In the widely used method based on modal sensitivity, which is also called mode-matching, regularisation is ensured by an additional term in the objective function that involves prior knowledge about the structural parameters being identified [37,38]. Conditioning of the problem can be enhanced by proper selection of the measurement locations as well as of the number of sensors and measured vibration modes [39,40]. In the case of CV measurement, selection of the measured locations can be done even after the measurement session. Blachowski noticed also that the accuracy of the results and the sparsity of the solution were improved by using an additional constraint, which ensured that the parameters representing stiffness (and thus representing the unknown structural damage) could only decrease during the optimization process based on modal sensitivities [39]. Additionally, the unknown parameters are not identifiable if the corresponding parameterized structural members are not involved in the structural response. This happens, for example, if the vibration modes that participate in structural vibration do not involve strains of the elements that are to be identified [41].

The literature review shows that significant effort has been devoted to enhancing the performance of CV measurements. A similar trend can be observed in SHM and CV-SHM. Similarly, an increase in the number of studies that employ low-cost cameras in smartphones can be observed due to the growing potential of cost-effective measurements. However, to the knowledge of the authors, there are still only a limited number of works that discuss the influence of limitations and measurement errors of computer vision equipment on the accuracy of structural identification procedures. In particular, low-cost consumer cameras and their use in ill-conditioned identification problems with a limited number of measurement data are not fully investigated.

The present study investigates the influence of CV measurement uncertainties and equipment (a CMOS smartphone camera) limitations on the accuracy of: dynamic displacement estimation, identification of modal parameters and the model updating procedure. The paper is structured as follows. In Section 2, the investigated frame structure and the corresponding parameterized finite element (FE) model are described. Section 3 describes the methodology: the employed CV displacement estimation with the aid of a smartphone camera, identification of modal parameters, and modal-sensitivity-based identification of the unknown parameters that describe the stiffness of structural bolted connections. In Section 4, benchmark accelerometer-based modal parameters are described and compared with the CV measured data and the identified parameters, including the identification uncertainties. Section 5 discusses the results and applicability of the investigated methodology. The conclusions are summarised in Section 6.

## 2. Structure under Investigation

### 2.1. Laboratory-Scale Frame Structure with Bolted Connections

**Figure 2:**
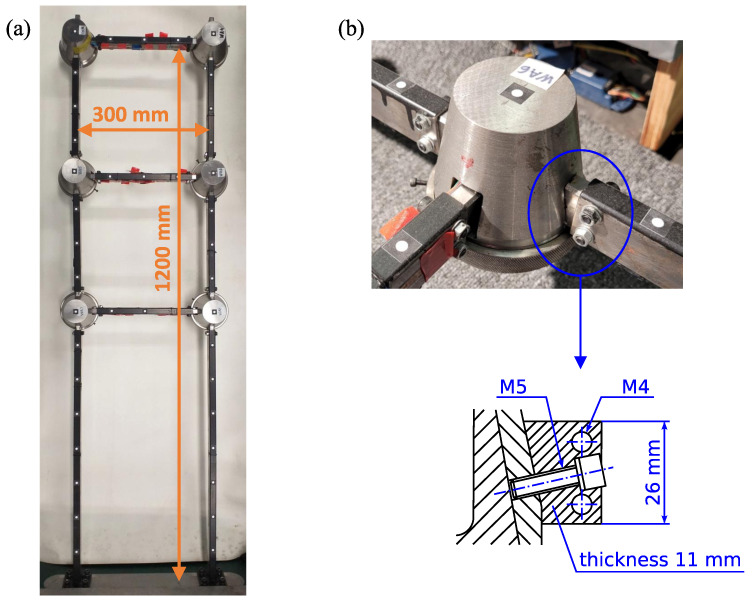
Photo of (**a**) investigated frame structure and (**b**) details of the bolted connections.

This subsection describes the investigated laboratory-scale frame structure shown in Figure 2a. It is equipped with six lockable joints, which are in the locked state during all experimental sessions. Thus, they are treated as rigid bodies. More details about these joints can be found in [42,43,44]. Steel rectangular beam profiles are connected with the joints via uncertain bolted connections whose stiffness is to be identified. These joints and bolted connections are shown in Figure 2b. Each beam profile has a rectangular hollow cross-section (RHS) of dimensions 15×30×2 mm. The frame structure is fixed in a steel support as shown in the bottom part of Figure 2a.

When all joints are in the locked state, the investigated structure does not exhibit any considerable nonlinear behaviour if the amplitude of motion is relatively low. Thus, a linear FE model and a linear system identification method are applied in this study. More details are shown in the next section.

### 2.2. Formulation of the Finite Element Model

A linear FE model $\left(M,K(\theta )\right)$ is proposed to reproduce the dynamic behaviour of the frame structure, where M is the constant mass matrix and K(θ) is the stiffness matrix, which depends on an unknown parameter vector $\theta ={\left[\begin{array}{cccc} \theta_{1} & \theta_{2} & \cdots & \theta_{N_{\theta }} \end{array}\right]}^{T}\in R_{+}^{N_{\theta }}$ as follows:

(1) $$K\left(\theta \right)=K_{0}+\sum_{t=1}^{N_{\theta }}\theta_{t}K_{t}.$$

**Figure 3:**
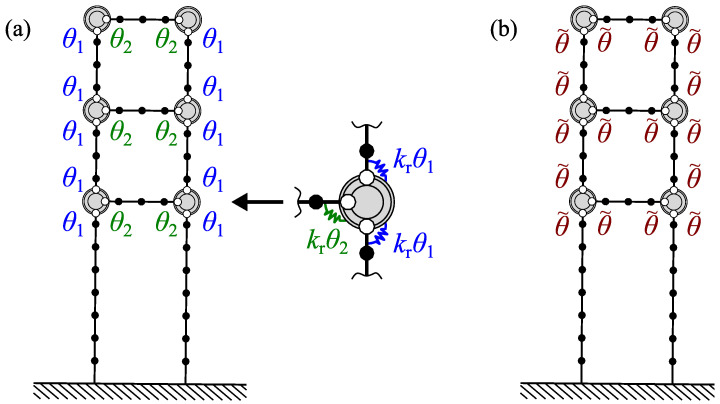
Mesh and parameterization of FE models: (**a**) independent parameterization for vertical ($\theta_{1}$) and horizontal ($\theta_{2}$) bolted connections and (**b**) common parameter $\tilde{\theta }$ for all bolted connections.

In Equation (1), the matrix $K_{0}$ represents the stiffness of the part of the structure whose properties are known, whereas the matrices $K_{t}$ represent the stiffness of the bolted connections. The FE mesh and the two considered parameterizations are shown in Figure 3. As shown in Figure 3, the behaviour of the bolted connection is represented by a semi-rigid node according to the formula:

(2) $$K_{t}=k_{r}l_{t}l_{t}^{T},$$

where $k_{r}$ is the nominal rotational stiffness, and

$$l_{t}={\left[\begin{array}{ccccccccccc} 0 & \cdots & 0 & 1 & 0 & \cdots & 0 & -1 & 0 & \cdots & 0 \end{array}\right]}^{T}$$

is the column transformation matrix that selects the rotational degrees of freedom (DOFs) involved in the semi-rigid node. The independent parameterization for the vertical and horizontal bolted connections follows from the fact that the conical surfaces of the joint to which the bolts are tightened have different curvatures (see Figure 2b), and thus their stiffness properties are also different, as shown in a previous work of the authors [45].

Figure 3a shows a parameterization that allows identification of two different stiffness parameters: $\theta_{1}$ for the bolted connections of the longitudinal beams (vertical bolted connections in Figure 2a and Figure 3a) and $\theta_{2}$ for the bolted connections of the transversal beams (horizontal bolted connections). Figure 3b shows a simplified parameterization where all bolted connections are assumed to share the same stiffness parameter $\tilde{\theta }$.

Both FE models have 139 DOFs. In-plane beam elements with 6 DOFs based on Euler–Bernoulli beam theory are employed. Cubic shape functions are used. The joints are represented as rigid bodies by using appropriate offsets between the semi-rigid nodes. The mass matrix M is consistent according to the FE shape functions. The Young modulus of the beam material is $E=2.1\times 10^{11}$ Pa (steel), the nominal rotational stiffness of the semi-rigid node that represents the bolted connection is $k_{r}=10^{4}$ Nm/rad, the beam material density is ρ=7840 kg/m^3^, the mass of the joint is $m_{J}=1.86$ kg, and the mass moment of inertia of the joint is $I_{J}=0.0015$ kg m^2^.

## 3. Identification of Unknown Parameters Based on Measurements from Computer Vision Systems

**Figure 4:**
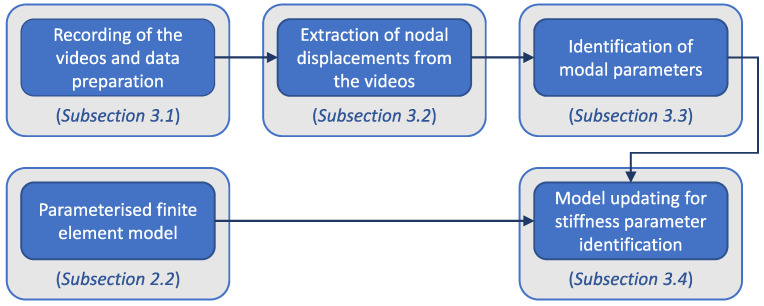
Flowchart of the investigated methodology.

In this section, all steps of the investigated CV methodology for identification of structural parameters are discussed. The order of the steps is presented in Figure 4 together with the references to the respective subsections.

### 3.1. Smartphone Camera with CMOS Sensor and Recorded Videos

In the present study, a Samsung Galaxy S20 FE smartphone equipped with a high-speed camera based on CMOS technology is used to register structural behaviour. The technical data of the camera are listed in Table 1. Among the three available recording modes, “Slow motion” (240 Hz) is selected because “Super slow motion” (960 Hz) provides lower resolution and allows recording of only 1 s long movies. Hence, the extracted lower-order modes would be of a lower accuracy.

**Figure 5:**
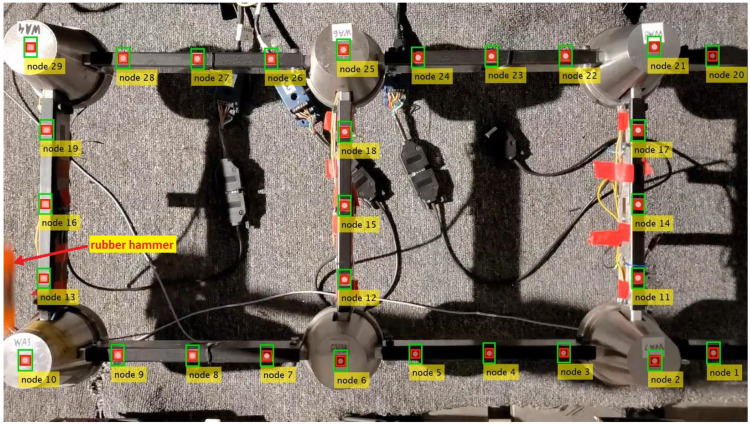
Example of the video frame registered by the smartphone camera accompanied by the numeration of the nodes to be tracked, manually selected templates (red rectangles), ROIs (green rectangles) and the location of the unmeasured impact excitation by a rubber hammer.

Aiming at achieving a trade-off between the largest field of view possible as required for multi-point measurements and satisfactory resolution, the smartphone is set to capture artificial targets located between the middle and the tip end of the structure. The distance from the smartphone to the structure is not measured. The recorded part of the structure with the manually selected templates around each measurement location (“node”) and the automatically generated ROIs is shown in Figure 5. The ROIs are generated by adding margins to the templates that are greater in the direction of the predominant vibration. There are 29 nodes located every 75 mm along the beams. A zoomed view of the artificial targets is shown in Figure 2b. A halogen lamp is used during the measurements to provide good illumination conditions.

Two depths of the image plane can be distinguished: one for the artificial targets located on the beams and another one for the targets on the joints (compare Figure 2 and Figure 5). Thus, two scaling factors are determined from the known physical and pixel dimensions of the structure: $\kappa_{1}=0.3916$ mm/px and $\kappa_{2}=0.3693$ mm/px for the nodes located on the beams and the joints, respectively. Four videos are recorded without changing the smartphone location; hence, the scaling factors remain the same for all the videos.

### 3.2. Algorithm Applied for Computer-Vision-Based Measurement

**Figure 6:**
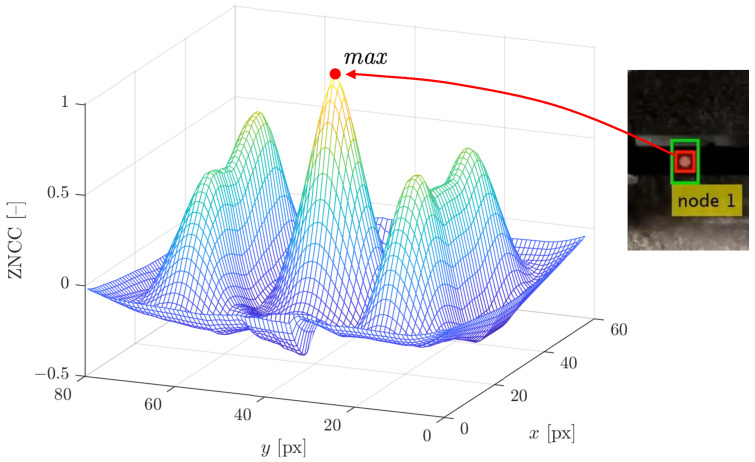
Example of the plot of the ZNCC function for Node 1.

In the present study, CV measurements are performed in laboratory conditions with good illumination during the experiments. Changes in the illumination and the scale can be considered negligible in the provided measurement conditions; hence, the robustness of the feature-based template matching with respect to these sources of error is not necessary. Thus, due to its high accuracy, the area-based template matching method is employed. The template is matched with the ROI by maximization of the ZNCC [12]:

(3) $$\mathrm{ZNCC}\left(x,y\right)=\frac{\sum_{\left(\zeta ,\eta \right)\in U_{T}}\left\{\left[T\left(\zeta ,\eta \right)-\bar{T}\right]\left[R\left(\zeta +x,\eta +y\right)-{\bar{R}}_{T}\left(x,y\right)\right]\right\}}{\sqrt{\sum_{\left(\zeta ,\eta \right)\in U_{T}}{\left[T\left(\zeta ,\eta \right)-\bar{T}\right]}^{2}}\sqrt{\sum_{\left(\zeta ,\eta \right)\in U_{T}}{\left[R\left(\zeta +x,\eta +y\right)-{\bar{R}}_{T}\left(x,y\right)\right]}^{2}}},$$

where (x,y) is the position of the template within the ROI, $U_{T}$ denotes the set of the points that represent the pixel locations in the local coordinates of the template, T(ζ,η) is the pixel intensity of the template at the point (ζ,η), $\bar{T}$ is the mean template pixel intensity, R(ζ+x,η+y) is the pixel intensity of the ROI at the point (ζ+x,η+y) in the current video frame, and ${\bar{R}}_{T}\left(x,y\right)$ is the mean of the pixel intensities of the ROI within the area currently overlapping with the template. An example of the ZNCC function is shown in Figure 6. The distinguishable peak corresponds to the found position of the artificial target.

To achieve subpixel precision, the ZNCC function is interpolated with cubic spline functions at a mesh 16 times denser than the query points. The displacements are extracted not in real time, but by postprocessing the recorded videos, and hence the estimation accuracy is of a higher priority than the computational time. Due to many local maxima of the ZNCC function, the global maximum is found by an exhaustive search (Figure 6).

### 3.3. Identification of Modal Parameters

The SSI-DATA method is used to identify the state space model of the structure. Subsequently, stabilization diagrams are used to identify the natural frequencies and the mode shapes for each recorded video. Finally, the identified modal parameters from all the recorded videos are averaged.

For a particular recorded video, the stable poles of the identified modes are found with the following criteria:

(4) $$\left\{\begin{array}{c} \frac{\left|{\hat{f}}_{n}^{\left(m\right)}-{\hat{f}}_{n-1}^{\left(m\right)}\right|}{{\hat{f}}_{n-1}^{\left(m\right)}}\leq \varepsilon_{f}\\ 1-\mathrm{MAC}\left({\hat{\phi }}_{n}^{\left(m\right)},{\hat{\phi }}_{n-1}^{\left(m\right)}\right)\leq \varepsilon_{\phi }\\ \frac{\left|{\hat{\zeta }}_{n}^{\left(m\right)}-{\hat{\zeta }}_{n-1}^{\left(m\right)}\right|}{{\hat{\zeta }}_{n-1}^{\left(m\right)}}\leq \varepsilon_{\zeta } \end{array}\right.$$

In the above system of inequalities, ${\hat{f}}_{n}^{\left(m\right)}$, ${\hat{\phi }}_{n}^{\left(m\right)}\in R^{N_{o}}$ and ${\hat{\zeta }}_{n}^{\left(m\right)}$ are the identified *m*th natural frequency, mode shape and the modal damping coefficient for the model order *n*, respectively; $N_{o}$ is the number of the measured outputs (DOFs); MAC(·,·) is the modal assurance criterion; and $\varepsilon_{f}$, $\varepsilon_{\phi }$ and $\varepsilon_{\zeta }$ are certain preselected thresholds for the natural frequencies, mode shapes and modal damping coefficients, respectively, which typically have values of $\varepsilon_{f}=0.01$, $\varepsilon_{\phi }=0.02$ and $\varepsilon_{\zeta }=0.05$.

The modal parameters are calculated as the mean values:

(5) $${\hat{f}}^{\left(m\right)}=\frac{1}{\left|S_{m}\right|}\sum_{n\in S_{m}}{\hat{f}}_{n}^{\left(m\right)},\;{\hat{\phi }}^{\left(m\right)}=\frac{1}{\left|S_{m}\right|}\sum_{n\in S_{m}}{\hat{\phi }}_{n}^{\left(m\right)},\;{\hat{\zeta }}^{\left(m\right)}=\frac{1}{\left|S_{m}\right|}\sum_{n\in S_{m}}{\hat{\zeta }}_{n}^{\left(m\right)},$$

where $S_{m}$ denotes the set of the stable poles for the *m*th mode. They are found among all stable poles as clustered, according to the following joint criteria:

(6) $$\left\{\begin{array}{c} \frac{\left|{\hat{f}}_{n}^{\left(m\right)}-{\hat{f}}_{n_{\min}}^{\left(m\right)}\right|}{{\hat{f}}_{n_{\min}}^{\left(m\right)}}\leq \varepsilon_{f}\kappa_{\varepsilon }\\ 1-\mathrm{MAC}\left({\hat{\phi }}_{n}^{\left(m\right)},{\hat{\phi }}_{n_{\min}}^{\left(m\right)}\right)\leq \varepsilon_{\phi }\kappa_{\varepsilon }\\ \frac{\left|{\hat{\zeta }}_{n}^{\left(m\right)}-{\hat{\zeta }}_{n_{\min}}^{\left(m\right)}\right|}{{\hat{\zeta }}_{n_{\min}}^{\left(m\right)}}\leq \varepsilon_{\zeta }\kappa_{\varepsilon }\\ \left|S_{m}\right|\geq N_{S} \end{array}\right.,$$

where $n_{\min}$ denotes the lowest stable pole for the *m*th mode, $\kappa_{\varepsilon }$ is an additional preselected coefficient, and $N_{S}$ is the preselected minimal number of stable poles that satisfy the remaining conditions in the system of inequalities (6). The values of $\kappa_{\varepsilon }$ and $N_{S}$ are selected by the trial-and-error method. Typically, $\kappa_{\varepsilon }$ is close to one, whereas $N_{S}$ is between 5 and 20.

The stabilization diagrams obtained with a smartphone camera can be highly contaminated, and thus, clustering of the stable poles according to system of inequalities (6) may result in a repeated occurrence of the same mode. In other words, the poles that should belong to a single cluster might be separated into several closely located clusters. This is resolved by matching multiple modes according to the criterion

(7) $$\mathrm{MAC}\left({\hat{\phi }}^{\left(m_{i}\right)},{\hat{\phi }}^{\left(m_{j}\right)}\right)-\frac{1}{2}\frac{{\left({\hat{\lambda }}^{\left(m_{i}\right)}-{\hat{\lambda }}^{\left(m_{j}\right)}\right)}^{2}}{{\hat{\lambda }}^{\left(m_{i}\right)}}\geq \alpha_{\mathrm{MAC}},$$

where ${\hat{\lambda }}^{\left(m\right)}={\left(2\pi {\hat{f}}^{\left(m\right)}\right)}^{2}$ is the *m*th identified system eigenvalue, and $\alpha_{\mathrm{MAC}}$ is a preselected threshold of a typical value greater than or equal to 0.9. Mode matching can also be performed without the additional term that quantifies the eigenvalues. Subsequently, the modal parameters are calculated once again according to Equation (5), but the set $S_{m}$ is enlarged to include all the stable poles of the modes matched with the criterion described in Inequality (7).

### 3.4. Identification of Stiffness Parameters

Stiffness parameters are found with the modal-sensitivity-based method by solving the optimization problem $\left(P_{1}\right)$:

$$\left(P_{1}\right)\;\;\begin{array}{cccc} \; & \mathrm{find}\; & \; & \theta \\ \; & \mathrm{to}\;\mathrm{minimize}\; & \; & F(\theta ) \end{array}$$

The objective function for the above optimization problem is defined as follows:

(8) $$F\left(\theta \right)=e_{M}^{T}\left(\theta \right)\sum_{M}^{-1}e_{M}\left(\theta \right)+e_{\theta }^{T}\left(\theta \right)\sum_{\theta }^{-1}e_{\theta }\left(\theta \right),$$

where the the first term represents the error between the identified and the numerical modal parameters of the updated FE model, whereas the second term quantifies the discrepancy between both models based on prior knowledge about the values of the parameters. The second term is also responsible for numerical regularisation. The quantities in Equation (8) are defined as follows:

(9) $$e_{M}=\left[\begin{array}{c} \hat{\lambda }\\ \hat{\phi } \end{array}\right]-\left[\begin{array}{c} \lambda_{\mathrm{num}}\left(\theta \right)\\ \phi_{\mathrm{num}}\left(\theta \right) \end{array}\right]$$

is the error between the identified and the numerical modal parameters collected into the vectors:

$$\begin{array}{cc} \hat{\lambda } & ={\left[\begin{array}{cccc} {\hat{\lambda }}^{\left(1\right)} & {\hat{\lambda }}^{\left(2\right)} & \cdots & {\hat{\lambda }}^{\left(N_{m}\right)} \end{array}\right]}^{T}\in R_{+}^{N_{m}},\\ \hat{\phi } & ={\left[\begin{array}{cccc} {\hat{\phi }}^{(1)T} & {\hat{\phi }}^{(2)T} & \cdots & {\hat{\phi }}^{(N_{m})T} \end{array}\right]}^{T}\in R^{N_{m}N_{o}},\\ \lambda_{\mathrm{num}}\left(\theta \right) & ={\left[\begin{array}{cccc} \lambda_{\mathrm{num}}^{\left(k_{1}\right)}\left(\theta \right) & \lambda_{\mathrm{num}}^{\left(k_{2}\right)}\left(\theta \right) & \cdots & \lambda_{\mathrm{num}}^{\left(k_{N_{m}}\right)}\left(\theta \right) \end{array}\right]}^{T}\in R_{+}^{N_{m}},\\ \phi_{\mathrm{num}}\left(\theta \right) & ={\left[\begin{array}{cccc} c_{1}L\phi_{\mathrm{num}}^{(k_{1})T}\left(\theta \right) & c_{2}L\phi_{\mathrm{num}}^{(k_{2})T}\left(\theta \right) & \cdots & c_{N_{m}}L\phi_{\mathrm{num}}^{(k_{N_{m}})T}\left(\theta \right) \end{array}\right]}^{T}\in R^{N_{m}N_{o}}, \end{array}$$

where ${\hat{\lambda }}^{\left(m\right)}={\left(2\pi {\hat{f}}^{\left(m\right)}\right)}^{2}$ is the *m*th identified eigenvalue, $\lambda_{\mathrm{num}}^{\left(k_{m}\right)}\left(\theta \right)$ and $\phi_{\mathrm{num}}^{\left(k_{N_{m}}\right)}\left(\theta \right)$ denote the $k_{m}$th numerical eigenvalue and eigenvector, respectively, obtained from the FE model, and the index $k_{m}$ is found according to the mode-matching criterion:

(10) $$k_{m}=\arg\max_{k}\mathrm{MAC}\left(\phi_{\mathrm{num}}^{\left(k\right)}\left(\theta \right),\;{\hat{\phi }}^{\left(m\right)}\right).$$

The vector $\theta \in R^{N_{\theta }}$ collects the unknown parameters, where in this study $N_{\theta }=2$ (Figure 3a) or $N_{\theta }=1$ (Figure 3b), L is a Boolean matrix that selects the measured DOFs, and $c_{m}$ is the modal scale factor:

(11) $$c_{m}=\frac{{\hat{\phi }}^{(m)T}L\phi_{\mathrm{num}}^{\left(m\right)}}{{∥L\phi_{\mathrm{num}}^{\left(m\right)}∥}^{2}},$$

where ∥·∥ is the square norm, $\sum_{M}$ is the prior or the estimated covariance matrix of the measurement error,

$$e_{\theta }\left(\theta \right)=\theta -\theta_{0},$$

and $\theta_{0}$ is the assumed initial vector of the unknown parameters, while $\sum_{\theta }$ is the assumed prior covariance matrix of the unknown parameters. The matrices $\sum_{M}$ and $\sum_{\theta }$ are usually diagonal. If the matrix $\sum_{M}$ is not known, it can be assumed that

(12) $$\sum_{M}=\mathrm{diag}\left({\left[\begin{array}{cccccc} v_{\lambda }^{2}{\hat{\lambda }}^{(1)2} & \cdots & v_{\lambda }^{2}{\hat{\lambda }}^{(N_{m})2} & \frac{v_{\phi }^{2}{∥{\hat{\phi }}^{\left(1\right)}∥}^{2}}{N_{o}}1_{\phi }^{T} & \cdots & \frac{v_{\phi }^{2}{∥{\hat{\phi }}^{\left(N_{m}\right)}∥}^{2}}{N_{o}}1_{\phi }^{T} \end{array}\right]}^{T}\right),$$

where $v_{\lambda }$ and $v_{\phi }$ denote the expected coefficients of variation of the eigenvalue (or natural frequency) and the mode shape, respectively, and $1_{\phi }$ is the vector of ones of the same length as the mode shape. Typical values are $v_{\lambda }=0.01$ and $v_{\phi }=0.1$.

The optimization problem $\left(P_{1}\right)$ cannot be solved directly due to the nonlinear dependence of the modal parameters $\lambda_{\mathrm{num}}^{\left(k_{m}\right)}\left(\theta \right)$ and $\phi_{\mathrm{num}}^{\left(k_{N_{m}}\right)}\left(\theta \right)$ on the unknown parameters θ. Thus, the method proposed by Friswell and Mottershead that employs the first-order expansion of the Taylor series is used to find the solution of $\left(P_{1}\right)$ in an iterative manner [37,38]. In each iteration step, the objective function $\tilde{F}$ defined in Equation (13) is to be minimized:

(13) $$\begin{array}{c} \tilde{F}\left(\Delta \theta \right)={\left(S\left(\theta \right)\Delta \theta -e_{M}\left(\theta \right)\right)}^{T}\sum_{M}^{-1}\left(S\left(\theta \right)\Delta \theta -e_{M}\left(\theta \right)\right)\\ \;\;\;\;\;\;\;\;\;\;\;\;\;+{\left(\theta +\Delta \theta -\theta_{0}\right)}^{T}\sum_{\theta }^{-1}\left(\theta +\Delta \theta -\theta_{0}\right), \end{array}$$

where Δθ is the increment of the unknown parameter vector θ at the current iteration step, and

(14) $$S\left(\theta \right)=\left[\begin{array}{ccc} \frac{\partial \lambda_{\mathrm{num}}^{\left(k_{1}\right)}}{\partial \theta_{1}} & \cdots & \frac{\partial \lambda_{\mathrm{num}}^{\left(k_{1}\right)}}{\partial \theta_{N_{\theta }}}\\ \vdots & \; & \vdots \\ \frac{\partial \lambda_{\mathrm{num}}^{\left(k_{N_{m}}\right)}}{\partial \theta_{1}} & \cdots & \frac{\partial \lambda_{\mathrm{num}}^{\left(k_{N_{m}}\right)}}{\partial \theta_{N_{\theta }}}\\ c_{1}L\frac{\partial \phi_{\mathrm{num}}^{\left(k_{1}\right)}}{\partial \theta_{1}} & \cdots & c_{1}L\frac{\partial \phi_{\mathrm{num}}^{\left(k_{1}\right)}}{\partial \theta_{N_{\theta }}}\\ \vdots & \; & \vdots \\ c_{N_{m}}L\frac{\partial \phi_{\mathrm{num}}^{\left(k_{N_{m}}\right)}}{\partial \theta_{1}} & \cdots & c_{N_{m}}L\frac{\partial \phi_{\mathrm{num}}^{\left(k_{N_{m}}\right)}}{\partial \theta_{N_{\theta }}} \end{array}\right]$$

is the modal sensitivity matrix. In this study, S(θ) has two columns or only one column based on the number of parameters considered (Figure 3). The eigenvalue sensitivities $\partial \lambda_{\mathrm{num}}^{\left(k_{m}\right)}/\partial \theta_{t}$ can be calculated as shown in [46], and mode shape sensitivities $\phi_{\mathrm{num}}^{\left(k_{m}\right)}/\partial \theta_{1}$ can be calculated as shown in [47].

From Equation (13), it follows that the unknown parameters can be updated at the *i*th iteration step as follows:

(15) $$\begin{array}{cc} \theta_{i+1} & =\theta_{i}+s_{\theta }\Delta \theta =\\ \; & =\theta_{i}+s_{\theta }{\left(S^{T}\left(\theta_{i}\right)\sum_{M}^{-1}S\left(\theta_{i}\right)+\sum_{\theta }^{-1}\right)}^{-1}\left[S^{T}\left(\theta_{i}\right)\sum_{M}^{-1}e\left(\theta_{i}\right)-\sum_{\theta }^{-1}\left(\theta_{i}-\theta_{0}\right)\right], \end{array}$$

where $s_{\theta }$ is a scale factor that is preselected with the trial-and-error method to provide convergence of the optimization procedure.

It can be shown that the covariance matrix that describes the uncertainties of the unknown parameters θ can be expressed as shown in Equation (16):

(16) $$V_{\theta }={\left(S^{T}\sum_{M}^{-1}S+\sum_{\theta }^{-1}\right)}^{-1}.$$

It is worth noticing that the variances $v_{ii}$ on the diagonal of $V_{\theta }$ do not describe the bias error resulting from systematic measurement errors or an incorrect selection of the parameters (or a class) of the model. Thus, a comparison of only the variances of the unknown parameters estimated using different classes of FE models can be misleading due to the bias–variance trade-off problem. More complex models usually have a lower bias, but they are also characterised by higher variances.

## 4. Comparison of Dynamic Displacements and Identified Parameters

In this section, the results of the methodology described in previous sections based on CV measurement with the aid of a smartphone camera are elaborated. First, the benchmark data based on accelerometers are described. Next, the accuracy of CV measurement with the aid of a smartphone camera is demonstrated. Subsequently, the identified modal data obtained with the smartphone camera and accelerometers are compared. Finally, model updating based on these two data sets is performed, and the results are compared.

**Figure 7:**
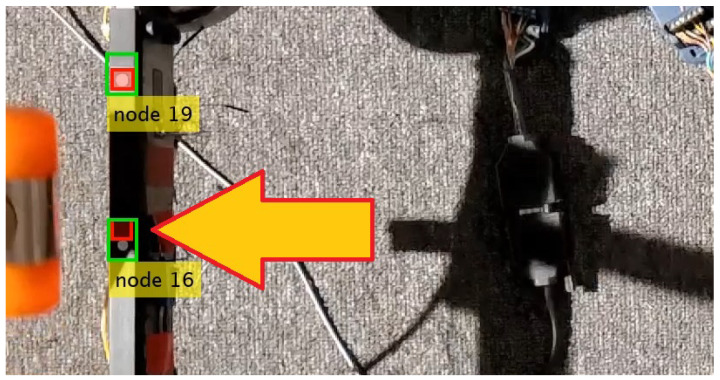
Example of the template mismatch caused by the shadow of the rubber hammer.

During the tests, Nodes 13, 16 and 19 were rejected due to the fact that the retraction of the rubber hammer after the hit causes contamination of the measurement by the hammer’s shadow. This phenomenon is demonstrated in Figure 7. The other tested feature-based template matching methods, e.g., orientation code matching (OCM) and KLT, exhibit a similar lack of robustness with respect to this perturbation, as in the ZNCC-based method. Thus, 26 nodes are finally available for CV measurement, and the identified state space has 52 DOFs.

In this section, accelerometer-based identified modal parameters are marked with the subscript “acc”, e.g., ${\hat{\lambda }}_{\mathrm{acc}}^{\left(m\right)}$ and ${\hat{\phi }}_{\mathrm{acc}}^{\left(m\right)}$, whereas the CV parameters are marked with the subscript “cv”, e.g., ${\hat{\lambda }}_{\mathrm{cv}}^{\left(m\right)}$ and ${\hat{\phi }}_{\mathrm{cv}}^{\left(m\right)}$.

### 4.1. Reference Data Obtained with Accelerometers

Accelerometer-based modal parameters were identified using the SSI method with the aid of an LMS-SCADAS system and the LMS Test.Lab software. All 26 locations of bidirectional accelerometers were selected to provide the results for comparison with the CV measurements. Accelerometers B&K 4507 B 004 were used in this study. Five experimental sessions were conducted, resulting in five data sets. The natural frequencies of these data sets are shown in Table 2. Data Set #1 was obtained using the impact testing technique, and only the natural frequencies were obtained. Data Sets #2 and #3 were obtained using the impact testing–roving hammer technique. Data Set #4 was obtained using the impact testing–roving accelerometer technique. Data Set #5 was obtained using modal shakers with the roving accelerometer technique. The modal shakers cannot work at frequencies below 60 Hz, and hence, the first two modes in the last data set were not identified.

**Figure 8:**
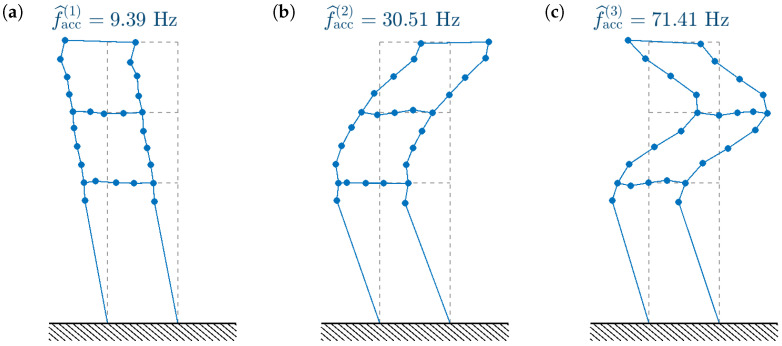
First three vibration modes identified with the aid of the bidirectional accelerometers (dots indicate accelerometer locations).

The modal parameters used later for model updating are calculated by averaging over all data sets; see Table 2 and Figure 8.

The normalised standard deviation $S_{m}^{\phi }$ presented in Table 2 is a metric of uncertainty of the *m*th mode shape, and it is calculated as follows:

(17) $$S_{m}^{\phi }=\frac{{\bar{\sigma }}^{\left(m\right)}}{\sqrt{\frac{1}{N_{o}}\sum_{i=1}^{N_{o}}{\hat{\phi }}_{i}^{(m)2}}},$$

where ${\hat{\phi }}_{i}^{\left(m\right)}$ is the *i*th element of the *m*th mode shape, and ${\bar{\sigma }}^{\left(m\right)}$ is the the mean standard deviation:

(18) $${\bar{\sigma }}^{\left(m\right)}=\frac{1}{N_{o}}\sum_{i=1}^{N_{o}}\sigma_{i}^{\left(m\right)},$$

while the standard deviation of the *i*th measured DOF of the *m*th mode shape $\sigma_{i}^{\left(m\right)}$ is estimated as follows:

(19) $$\sigma_{i}^{\left(m\right)}=\sqrt{\frac{1}{N_{d}-1}\sum_{d=1}^{N_{d}}{\left({\hat{\phi }}_{id}^{\left(m\right)}-{\hat{\phi }}_{i}^{\left(m\right)}\right)}^{2}}.$$

The physical sense of $S_{m}^{\phi }$ is that a substitution of $v_{\phi }$ in Equation (12) with $S_{m}^{\phi }$ results in the diagonal elements associated with the DOFs of the *m*th mode being equal to ${\left({\bar{\sigma }}^{\left(m\right)}\right)}^{2}$.

### 4.2. CV Measurement of Dynamic Displacements

In this subsection, the accuracy of CV measurement of dynamic displacement is demonstrated. The CV measurement of transversal displacement of Node 2 (Figure 5) is compared with the data obtained with the LDS, the accelerometer and their fusion. Hence, LDS Baumer 2016160/S14F and the digital oscilloscope Tektronix TDS 2004C are also used to register the time-domain data.

The vector containing the displacement time series u resulting from the data fusion is found by solving the optimization problem $\left(P_{2}\right)$ [48]:

$$\left(P_{2}\right)\;\;\begin{array}{cccc} \; & \mathrm{find}\; & \; & u\\ \; & \mathrm{to}\;\mathrm{minimize}\; & \; & J(u), \end{array}$$

where the objective function is defined as follows:

(20) $$J\left(\theta \right)=\frac{1}{2}{∥W\left(\mathrm{Du}-\Delta t^{2}a_{\mathrm{acc}}\right)∥}^{2}+\frac{\gamma^{2}}{2}{∥u-q_{\mathrm{LDS}}∥}^{2}$$

In Equation (20), W is a diagonal weighting matrix whose diagonal contains only ones and $1/\sqrt{2}$ in the first and last element; D is the second order differential operator matrix; Δt is the time step, equal here to 2 ms; $a_{\mathrm{acc}}$ is the time series of acceleration measured by the accelerometer; γ is a weighting coefficient, selected here to be equal to 0.4; and $q_{\mathrm{LDS}}$ is the displacement time series measured by the LDS. The problem $\left(P_{2}\right)$ is solved directly:

(21) $$u={\left(D^{T}W^{2}D+\gamma^{2}I\right)}^{-1}\left(D^{T}W^{2}a_{\mathrm{acc}}\Delta t^{2}+\gamma^{2}q_{\mathrm{LDS}}\right).$$

**Figure 9:**
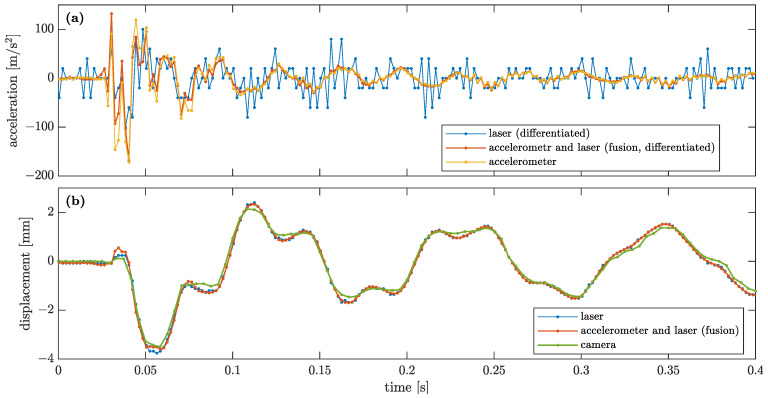
Comparison of (**a**) accelerations and (**b**) corresponding displacements of Node 2 obtained by various types of sensing of the structural dynamics.

The result is visualised in Figure 9 along with the results obtained from the accelerometer, the LDS and the smartphone camera. It is evident that the differentiated LDS-based data are much noisier than the accelerations measured directly by the accelerometer. On the other hand, the displacement obtained from the data fusion stays in good agreement with both the LDS-based data and the accelerometer-based data; see Figure 9a. All the measured displacements compared in Figure 9b are in good agreement. The displacement estimated using the smartphone camera seems to be slightly more distorted than the other results, which are almost the same; however, the error level is still at a very satisfactory level. The error of the CV measurement with respect to the fused results can be expressed as:

(22) $$e_{\mathrm{cv}}=\frac{\mathrm{std}(q_{\mathrm{cv}}-u)}{\mathrm{std}(u)}=10.82\%,$$

where std(·) means the standard deviation, and $q_{\mathrm{cv}}$ is the time series of the CV displacement. The analogously calculated error for the LDS-based data is

(23) $$e_{\mathrm{LDS}}=\frac{\mathrm{std}(q_{\mathrm{LDS}}-u)}{\mathrm{std}(u)}=5.14\%.$$

The errors $e_{\mathrm{cv}}$ and $e_{\mathrm{LDS}}$ are calculated for a time period of 1 s duration. The time series u and $q_{\mathrm{LDS}}$ are interpolated onto the time steps of $q_{\mathrm{cv}}$ with the use of spline functions to allow appropriate calculations in Equation (22). The error of the CV measurement is two times greater than that of LDS.

**Figure 10:**
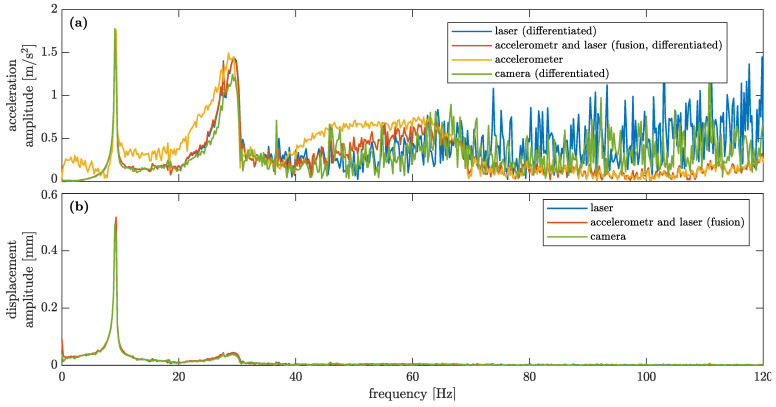
Comparison of amplitude spectra of (**a**) accelerations and (**b**) corresponding displacements of Node 2 obtained for various types of sensing of the structural dynamics.

Amplitude spectra for accelerations and displacements of Node 2, as obtained with various sensing techniques, are shown in Figure 10a and b, respectively. For calculation of amplitude spectra, signals of a duration of 4.5 s are taken into account with their original sampling frequencies, i.e., 500 Hz for the accelerometer, LDS and their fusion, and 240 Hz for the smartphone camera. It is evident that both the acceleration and the displacement amplitude spectra are in a good agreement in the vicinity of the first two natural frequencies. The LDS and CV accelerations become noisier above the frequency of 40 Hz due to the fact that they are calculated from displacements that are very small in this frequency range (higher-order modes need more energy to be excited, and they are usually characterised by significantly higher damping factors than lower-order modes). The fused acceleration data exhibit a trade-off expressed by the weighting coefficient γ; see Equation (20). In the frequency range 40–65 Hz, the fused data have values between those of the accelerometer and the LDS-based (and CV) data, whereas above this range they are in good agreement with the accelerometer-based data, which are less noisy.

The data shown in Figure 10 confirm that only three in-plane vibration modes can be identified below the Nyquist frequency of the CV measurement (120 Hz) because no other mode more is demonstrated in the acceleration spectra. The next in-plane mode of the investigated structure is present at 226 Hz [45].

The data fusion results are close to both input accelerations as measured by the accelerometer and as obtained from the LDS-based displacements. This suggests that the accelerometer-based measurement provides reliable benchmark data for identification of modal parameters (Section 4.3) and, thereupon, for model updating (Section 4.4).

### 4.3. Identified Natural Frequencies and Mode Shapes

In this subsection, modal parameters are described and discussed, as identified from the CV measurement data using the SSI-DATA method and the stabilization diagrams. Four videos have been recorded. A recording duration of 8.5 s is selected for each video, and it includes nearly 80 periods of vibration of the first mode.

The stabilization diagram is constructed as described in Section 3.3 with the model orders ranging from 1 to 104, which is twice the number of the measured outputs. The coefficient values $\varepsilon_{f}=0.01$, $\varepsilon_{\phi }=0.02$, $\varepsilon_{f}=0.05$, $\kappa_{\varepsilon }=1.5$, $N_{S}=5$ and $\alpha_{\mathrm{MAC}}=0.9$ are used. Among the four recorded videos, only one allows three vibration modes to be identified. The other three videos allow only the first two vibration modes to be identified.

**Figure 11:**
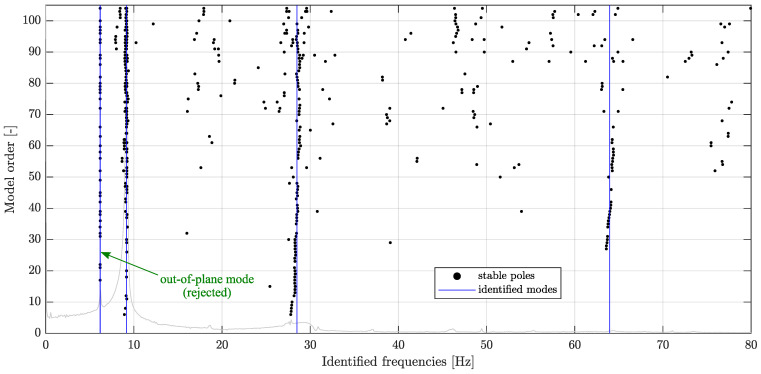
Stabilization diagram with the identified modes, including one rejected out-of-plane mode, as obtained in the fourth video.

An example of the stabilization diagram for the fourth video is shown in Figure 11. The stabilization diagrams also include out-of-plane modes, which are rejected because only the in-plane modes are considered in this study, as shown in Figure 11. Since the displacements in the third dimension are not measured by the smartphone camera, these modes are selected according to the MAC criterion between the CV modes and the accelerometer-based modes, which was expected to be higher than 0.7. Generally, the calculated numerical modes can be also used if accelerometer-based modal parameters are not available.

It is evident that the use of methods intended to cluster stable poles and to reject spurious modes, as described in Section 3.3, allows for obtaining clearly demonstrated modal data, as shown in Figure 11.

The CV natural frequencies and the calculated uncertainties are listed in Table 3. The final values of the identified modal parameters ${\hat{f}}_{\mathrm{cv}}^{\left(m\right)}$ and ${\hat{\phi }}_{\mathrm{cv}}^{\left(m\right)}$, m=1,2,3 are calculated as the mean of the data available in all four videos. The uncertainties are calculated analogously to the accelerometer-based modal parameters; see Table 2 and Equations (17)–(19).

The uncertainties in the first two CV modal parameters are smaller than those of the accelerometer-based modal parameters. Due to a possible systematic error, this does not necessarily mean that the CV measurement is more accurate, but it demonstrates that the CV data are more consistent. In fact, this error can be characterised as the bias error: the natural frequencies of the CV-identified modes seem to be underestimated, especially the third one. This is clearly demonstrated in Table 4. The underestimation error increases nearly proportionally to the identified natural frequency. Despite the fact that for the third mode, the MAC has the lowest value of 0.93, it still remains at a satisfactory level above 0.9 for all the vibration modes.

**Figure 12:**
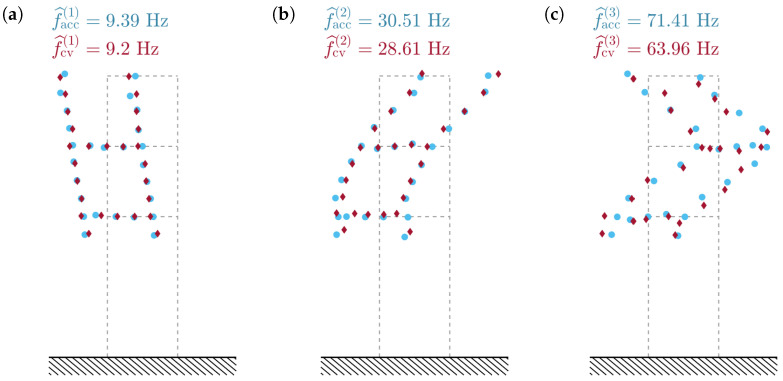
Comparison of the identified mode shapes obtained with accelerometers (blue dots) and smartphone camera (claret dots).

A comparison of the mode shapes obtained with the accelerometers and the smartphone camera is shown in Figure 12. The high MAC values are reflected in the high similarity between the mode shapes identified using both methods. The third CV mode shape seems the noisiest; however, the mode shape is still properly reflected and well-correlated with the accelerometer-based result.

### 4.4. Identified Stiffness Parameters

This subsection presents and compares the results of model updating for accelerometers and CV data. The weighting matrix $\sum_{M}^{-1}$ is selected in accordance with Equation (12) with the parameters $v_{\lambda }=0.01$ and $v_{\phi }=0.05$ both for CV- and accelerometer-based identified modal parameters. These values correspond with the COVs and NSDs shown in Table 2 and Table 3, and they are not far from typical values. Pursuing the COVs and NSDs estimated from the available data sets makes comparison of the results difficult, since the COV and NSD for the third mode obtained with the smartphone camera are not available.

The initial values of the unknown parameters in $\theta_{0}$ are assumed to be equal to one. The prior covariance matrix $\sum_{\theta }$ is assumed to be diagonal, with all elements on the diagonal equal to the prior variance $s_{\theta }^{2}=0.25$. Consequently, assuming Gaussian distribution, each unknown parameter is within the interval [0,2] with a probability of 95%.

The scaling factor $\kappa_{\theta }=0.4$ is selected with the trial-and-error method. The model updating procedure is stopped when all unknown parameters differ from the corresponding values in the previous iteration by less than one percent. The model updating procedure is performed for three cases: when only the first mode (natural frequency and mode shape) is identified and available, when the first two identified modes are available, and when all three modes are available.

**Figure 13:**
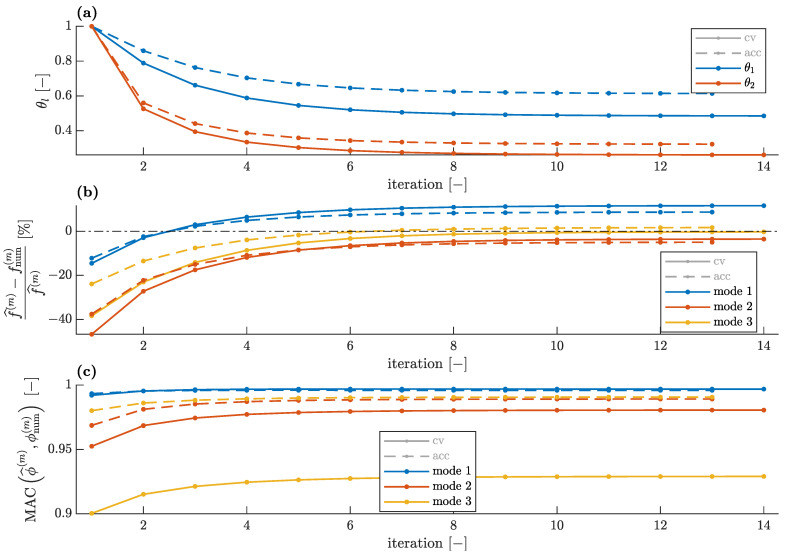
Comparison of convergences of model updating employing accelerometer and CV modal data for two unknown parameters: (**a**) unknown parameters, (**b**) relative natural frequency error and (**c**) modal assurance criterion.

**Figure 14:**
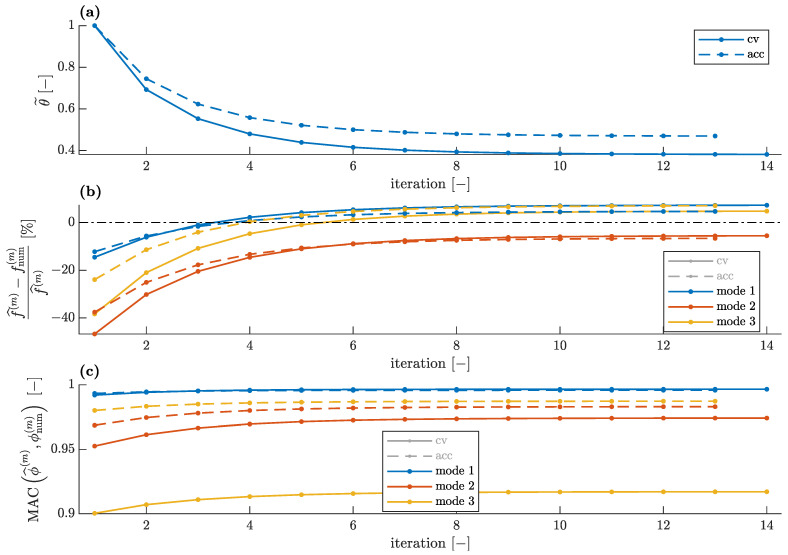
Comparison of convergences of model updating employing accelerometer and CV modal data for single unknown parameter: (**a**) unknown parameter, (**b**) relative natural frequency error and (**c**) modal assurance criterion.

Comparison of the convergences for the CV- and accelerometer-based data when all three identified modes are available and two stiffness parameters are used (see Figure 3a) is shown in Figure 13. For a single stiffness parameter (see Figure 3b), the corresponding convergences are shown in Figure 14.

For both CV- and accelerometer-based data, the values of the unknown parameters converge without any numerical difficulties. The errors between the identified and numerical modal parameters decrease for the CV- and accelerometer-based data as well as for the parameterizations with two and the single unknown parameter.

The errors in the numerical modal parameters obtained for the CV measurement data are greater than the errors obtained with the accelerometer-based data, both for the single unknown parameter and for the two unknown parameters used in model updating. This is due to two reasons: The first is that the initial FE model overestimated natural frequencies, and thus, the lower natural frequencies identified with the smartphone camera increase this error. Hence, the discrepancy between the numerical and the CV-identified frequencies is greater than the corresponding discrepancy between the numerical and the accelerometer-based identified frequencies, as demonstrated in Figure 13b and Figure 14b. The second reason is that the CV measurement data are generally expected to have greater errors, as visible especially in Figure 12c. Thus, even for the updated FE model, the MAC values between the numerical and CV-identified mode shapes are lower than the corresponding values for the accelerometer-based mode shapes (Figure 13c and Figure 14c). Nevertheless, all MAC values remain at a satisfactory level above 0.9. Additionally, in the case of the CV data, the lower identified natural frequencies result in a slightly lower level of the unknown parameters (Figure 13a and Figure 14a).

**Figure 15:**
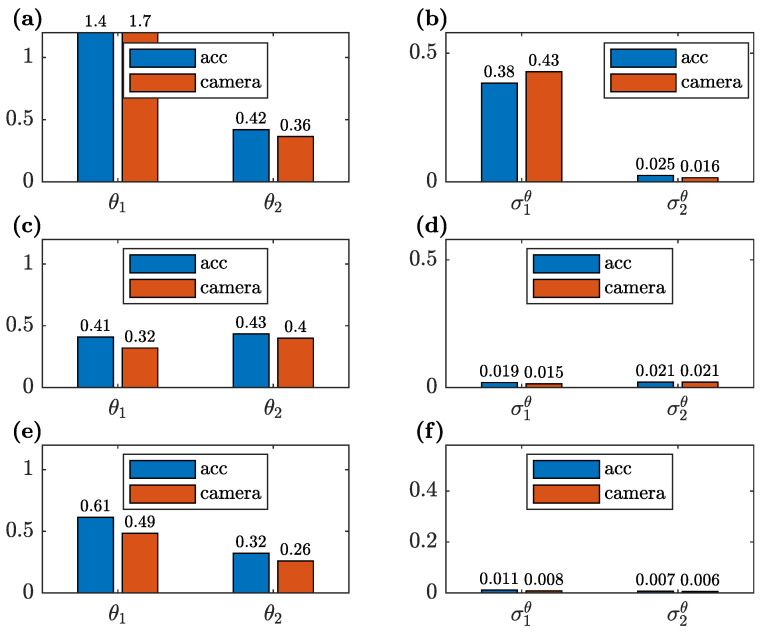
Comparison of the unknown parameters $\theta_{1}$ and $\theta_{2}$ of the updated FE model obtained from the CV- and accelerometer-based data and the corresponding expected standard deviations $\sigma_{1}^{\theta }$ and $\sigma_{2}^{\theta }$ for: (**a**,**b**) only the first identified mode available; (**c**,**d**) the two first identified modes available and (**e**,**f**) all three modes available for model updating.

**Figure 16:**
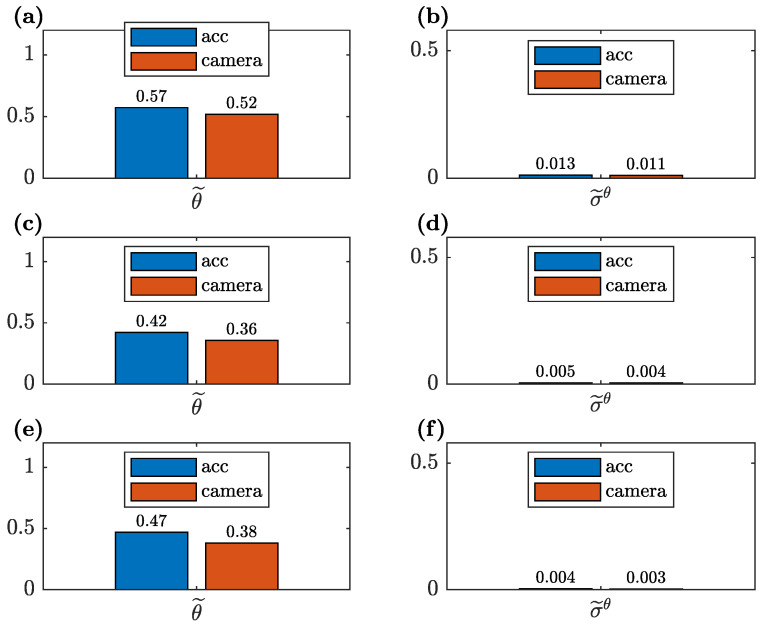
Comparison of the unknown parameter $\tilde{\theta }$ of the updated FE model obtained from the CV- and accelerometer-based data and the corresponding standard deviation ${\tilde{\sigma }}^{\theta }$ for: (**a**,**b**) only the first identified mode available; (**c**,**d**) the two first identified modes available and (**e**,**f**) all three modes available for model updating.

A comparison of the unknown parameters $\theta_{1}$ and $\theta_{2}$ for all three cases of the available measurement data, estimated based on the CV and accelerometer measurement data, together with the corresponding standard deviations, is shown in Figure 15. The analogous comparison for the reduced parameterization with a single parameter ${\tilde{\theta }}_{1}$ is shown in Figure 16. The estimated standard deviation $\sigma_{t}^{\theta }$ of the unknown parameter is calculated as the square root of the corresponding diagonal element of the matrix $V_{\theta }$; see Equation (16). To this end, the matrix $\sum_{M}$ is employed, which is calculated as shown in Equation (12) with $v_{\lambda }=0.01$ and $v_{\phi }=0.05$, since Equation (16) is true only if the weighting matrices in the model updating procedure are equal to the reciprocals of the corresponding covariance matrices (see Equation (8)). Such a covariance matrix gives information about parameter uncertainty for typical measurement variances.

It is evident that the results are dependent on the number of the available measured modes. Both for the CV- and accelerometer-based data, the unknown parameters have different values in each measurement data case. Simultaneously, the standard deviations of the unknown parameters are smaller with the increase of the available measurement data. These observations are visible for both considered parameterizations (two and a single unknown parameter). However, the single unknown parameter seems to be less sensitive to the amount of the measurement data, and it has a lower standard deviation than the corresponding results obtained for the two parameters $\theta_{1}$ and $\theta_{2}$. Especially, as shown in Figure 15a, the unknown parameter $\theta_{1}$, estimated when only the first identified mode is available, has a value significantly different than in the other data cases. This is due to the fact that a single mode provides an insufficient amount of information to precisely estimate two unknown parameters. In other words, the greater complexity of the model and a smaller amount of the available measurement data tilt the bias–variance trade-off towards increased variance. This is also visible in the corresponding significantly higher variance of this parameter (Figure 15b).

The parameter $\theta_{2}$ tends to be lower than $\theta_{1}$ (Figure 15a,e) due to the different curvature of the adjacent conical surfaces involved in the vertical and horizontal bolted connections (Figure 2b). However, for both parameters, the estimation accuracy strongly depends on the available measurement data. The differences between particular results of the model-updating procedure are higher than is suggested by the calculated standard deviations of these parameters.

The considerations above refer both to the CV- and accelerometer-based measurement data. Moreover, the number of available identified modes has a greater influence on the values of the unknown parameters than the errors in the CV identification of the modal parameters described in Section 4.3, including the significant bias error of the natural frequencies.

**Figure 17:**
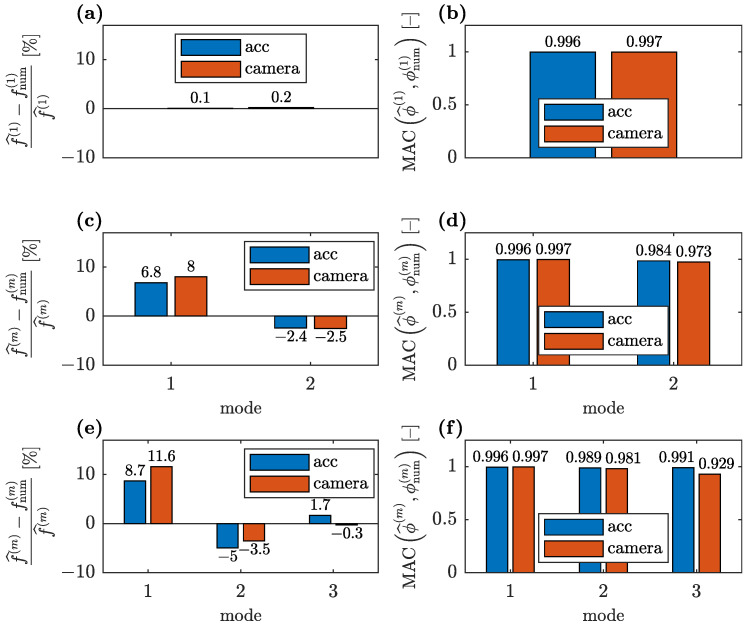
Comparison of the relative natural frequency error and MAC between the numerical and identified modal parameters (for parametrization with two unknown parameters $\theta_{1}$ and $\theta_{2}$) with the corresponding results for CV-identified modal parameters: (**a**,**b**) when only the first identified mode is available for model updating; (**c**,**d**) when two first modes are available; and (**e**,**f**) when all three identified modes are available.

**Figure 18:**
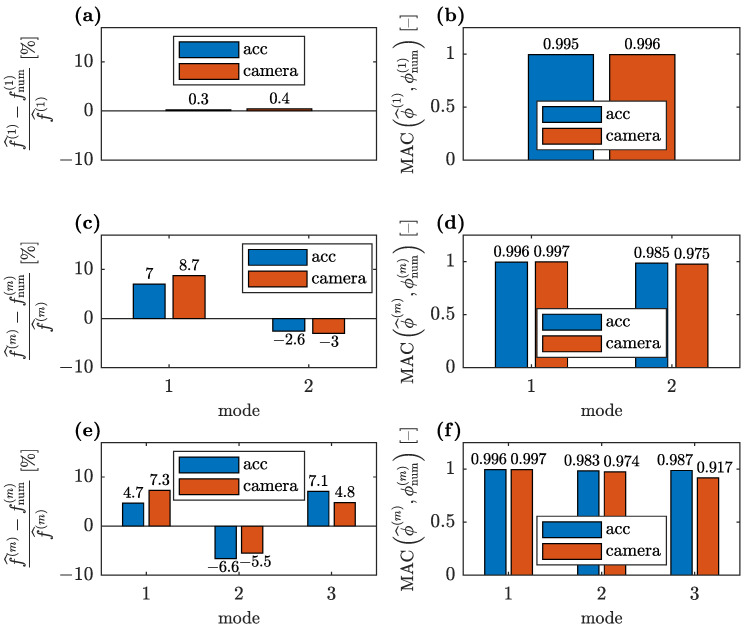
Comparison of the relative natural frequency error and MAC between the numerical and identified modal parameters (for parametrization with a single unknown parameter $\tilde{\theta }$) with the corresponding results for CV-identified modal parameters: (**a**,**b**) when only the first identified mode is available for model updating; (**c**,**d**) when two first modes are available; and (**e**,**f**) when all three identified modes are available.

A comparison of the error metrics between the numerical and accelerometer-based identified modal parameters with the corresponding error metrics obtained for the CV-identified parameters for various available identified modes when the FE model is updated with the two unknown parameters $\theta_{1}$ and $\theta_{2}$ is shown in Figure 17. An analogous comparison for the FE model parameterized with one parameter $\tilde{\theta }$ is shown in Figure 18.

It is evident that the errors between the numerical and identified modal parameters for the simplified parameterization of the FE model (Figure 18) are only slightly higher than for the parameterization with two parameters (Figure 17), whereas they provide significantly lower variances of unknown parameters and lower sensitivity to the availability of the modal parameters. For both parameterizations, all obtained relative errors of natural frequencies, except one (Mode 1 shown in Figure 17e) are below the level of 10%. Similarly, all MAC values are well above 0.9, which is a satisfactory result. For both parameterizations, the MAC calculated for the third CV-identified mode shape has the worst value due to the significant noise affecting this mode shape (Figure 12c).

## 5. Discussion of the Results

In this subsection, the obtained results are discussed in the context of their applicability both for real-world structures and laboratory experiments.

The third CV-identified mode has a natural frequency above 1/2 of the Nyquist frequency due to the limited sampling frequency of the smartphone camera. This results in considerable systematic error. The third mode shape also becomes noisy. However, real-world structures are often characterised by natural frequencies of much lower values; hence, they are less subject to estimation error when a camera with a limited sampling frequency is used. In [26], a camera mounted on a UAV and on a tripod recorded cable vibration with a sampling frequency of 60 Hz and a resolution of 3096 × 2160 px, and a sampling frequency 25 Hz and a resolution of 2048 × 2048 px, respectively. The natural frequencies equal to 1.03, 3.05 and 3.17 Hz obtained with the camera mounted on the tripod were in satisfactory agreement with accelerometer-based data obtained with a sampling frequency of 50 Hz. The camera mounted on the UAV measured the natural frequencies of higher-order modes to be 9.41, 10.43 and 11.43 Hz. Additionally, in this case, satisfactory agreement with accelerometer-based data was obtained despite the lower-order modes being omitted since they were affected by a low-frequency UAV motion during its hovering. These two tested cameras and accelerometers provided consistent estimated cable forces. The smartphone used in the present research can be set into the normal recording mode, which allows recording in 4 K resolution and with a sampling frequency of 60 Hz, i.e., the same as the UAV in [26]. The capability of the smartphone camera to measure low-frequency oscillations up to 2 Hz with a sampling frequency 30 Hz was also confirmed in [33], where a smartphone was proposed as a low-cost device to measure the interstory drift of buildings subjected to earthquakes. In the present study, the first two CV-identified vibration modes of the investigated structure (that are far from the Nyquist frequency of 120 Hz) are also in good agreement with the accelerometer-based identified modal parameters. It follows that consumer-grade electronics are suitable for measurement of flexible structures whose modes of interest are much lower than the Nyquist frequency. If this requirement is satisfied, the smartphone camera can be also used for 3D displacement measurement with the method proposed by Narazaki, since the projection of 2D displacements to 3D is a postprocessing procedure [9].

Regarding the parametric identification of the structural properties, in the investigated case, the number of the available modal parameters used in the optimization procedure affects the results more than the uncertainties resulting from CV measurement. A similar observation is given by Blachowski, who indicated that an increase in the number of measured modes is more profitable than an increase in the number of measurement locations [39]. This is one more argument in support of consumer-grade cameras being suitable for large-scale or flexible structures, which usually have small, lower-order natural frequencies, as more modes can be identified within the limited sampling frequency. The model updating method adopted in this study is widely accepted, and it can be used for various types of structures if the FE model of the structure is available. For example, again taking into account the work [26], the methodology proposed in the present paper could be implemented to monitor the cable condition. If only natural frequencies are to be identified, then the modal sensitivity matrix contains only the related rows, without the entries related to the mode shapes; see Equation (14). Only one unknown parameter scaling the stiffness is required for a particular cable for it to be sufficient to determine the cable condition. Hence, the problem would be well overdetermined. The methodology of monitoring the cable condition proposed in [26] does not require an FE model, but a methodology based on model updating would enable monitoring of not only several cables at once but also of all parts of the structure visible by the camera. As shown by Blachowski et al., modal-sensitivity-based model updating can be used with a relatively large number of unknown parameters. If still required, the number of the updated FEs (corresponding with the monitored structural members) can be reduced by an investigation of their influence on the modal parameters after calculation of the modal sensitivity matrix. Columns of this matrix that reveal a low influence on the natural frequencies (small eigenvalue derivatives) can be rejected, since this means that the corresponding elements do not transfer significant structural loads, and their monitoring is of lower importance. Including these aspects in the researched methodology may facilitate CV-SHM based on consumer-grade low-cost devices of large scale structures.

In recent times, machine and deep learning approaches in SHM have become more and more popular. Artificial neural networks can enhance the performance of SHM techniques, especially when the monitored structure is large-scale and exhibits a nonlinear relation between the damage and measured output [49]. The methods employed in the present study are suitable and efficient for linear problems. Large-scale FE models can be reduced with, e.g., the dynamic reduction method or the system equivalent reduction expansion process (SEREP) [37].

## 6. Conclusions

This study investigated the influence of the equipment limitations and errors of CV measurement on the identification of both modal parameters and unknown structural parameters that represented stiffness of structural bolted connections. The conclusions can be drawn as follows:

1. The employed consumer-grade smartphone camera with the “slow-motion” mode selected, full HD resolution and a sampling frequency of 240 Hz allowed us to estimate structural displacement with an error two times higher than that when using LDS (10.82% vs. 5.14%), where a fusion of LDS- and accelerometer-based data were taken as the benchmark.
2. The selected mode of recording allowed the first three vibration modes to be identified. The MAC between the CV- and the corresponding accelerometer-based identified mode shapes had satisfactory values: 0.99, 0.98 and 0.92. However, the identified natural frequency was underestimated: the first natural frequency (9.39 Hz) was underestimated with an error of 2.03%, whereas the third one (71.40 Hz) had an error of 10.43%. This error was recognised as systematic, since the variances of the CV-identified modal parameters were not higher than the accelerometer-based ones.
3. The unknown parameters representing the stiffness of the bolted connections were identified with the modal-sensitivity-based model updating technique. The parameters identified using CV measurements had slightly lower values than the ones based on accelerometers, which was due to the underestimated natural frequencies. However, it was shown that the number of identified modes taken into account had a significantly greater influence on the unknown parameters than the systematic error of the CV-identified natural frequency. Reduction of the number of unknown parameters reduced their sensitivity to the number of available measurement data due to a more profitable bias–covariance trade-off.
4. Despite the noticeable errors obtained with the use of the smartphone camera, it should be noted that the cost of consumer-grade equipment is significantly lower than that of accelerometers and a data-acquisition system with integrated software. Moreover, a camera allows performance of “by hand” and multiple-point measurements at once.

The results provide insight into the expected error types and levels, not only for the directly measured quantities but also for the parameters identified in structural dynamics, when using low-cost consumer-grade electronics.

## Figures and Tables

**Table 1:** Technical data of Samsung Galaxy S20 FE speed camera.

System Component	Parameter	Value
Sensor	Technology	CMOS
	Model	Sony IMX555
	Dimensions	7.26 × 5.44 mm
	Resolution	4032 × 3024 px (12.2 Mpx)
	Pixel size	1.8 µm
	Chroma	RGB
Recording mode	Normal	60 Hz,
(sampling frequency,		3840 × 2160 px (4K),
resolution, duration)		Unlimited
	Slow motion	240 Hz,
		1920 × 1080 px (Full HD),
		Unlimited
	Super slow motion	960 Hz,
		1280 × 720 px (HD),
		1 s
Optics	Focal length	5.4 mm
	Reference focal length ^1^	26.8 mm
	Maximum aperture	*f*/1.8
	Horizontal FoV ^2^	67.8°
	Vertical FoV	53.5°

^1^ Reference focal length recalculated for reference sensor size 35 mm used for comparison with other cameras; ^2^ Field of view.

**Table 2:** Identified modal parameters based on accelerometers.

IP Mode	Identified Freq. ${\hat{f}}_{\mathrm{acc}\;d}^{\left(m\right)}$ (Hz) for Each Dataset *d*					Mean	COV ^1^	NSD ^2^
*m* [−]	#1	#2	#3	#4	#5	${\hat{f}}_{\mathrm{acc}}^{\left(m\right)}$ (Hz)	$V_{m}^{f}$ (%)	$S_{m}^{\phi }$ (%)
1	9.39	9.49	9.46	9.23	—	9.39	1.24	5.34
2	30.41	30.6	30.5	30.54	—	30.51	0.26	5.11
3	70.4	71.55	71.55	70.98	72.52	71.4	1.1	5.13

^1^ Coefficient of variation; ^2^ Normalized standard deviation for corresponding mode shapes.

**Table 3:** Identified modal parameters based on computer vision (smartphone camera).

IP Mode	Identified Freq. ${\hat{f}}_{\mathrm{cv}\;v}^{\left(m\right)}$ (Hz) for Each Video *v*				Mean	COV ^1^	NSD ^2^
*m* [−]	#1	#2	#3	#4	${\hat{f}}_{\mathrm{cv}}^{\left(m\right)}$ (Hz)	$V_{m}^{f}$ (%)	$S_{m}^{\phi }$ (%)
1	9.16	9.27	9.23	9.15	9.20	0.58	1.99
2	28.54	28.79	28.62	28.50	28.61	0.45	4.09
3	—	—	—	63.96	63.96	—	—

^1^ Coefficient of variation; ^2^ Normalized standard deviation for corresponding mode shapes.

**Table 4:** Comparison of CV- and accelerometer-based identified modal parameters.

*m* [–]	${\hat{f}}_{\mathrm{acc}}^{\left(m\right)}$ (Hz)	${\hat{f}}_{\mathrm{cv}}^{\left(m\right)}$ (Hz)	$\frac{{\hat{f}}_{\mathrm{cv}}^{\left(m\right)}-{\hat{f}}_{\mathrm{acc}}^{\left(m\right)}}{{\hat{f}}_{\mathrm{acc}}^{\left(m\right)}}$ (%)	$\mathrm{MAC}\left({\hat{\phi }}_{\mathrm{acc}}^{\left(m\right)},{\hat{\phi }}_{\mathrm{cv}}^{\left(m\right)}\right)$ [–]
1	9.39	9.20	−2.03	0.99
2	30.51	28.61	−6.24	0.98
3	71.40	63.96	−10.43	0.93

## Data Availability

Upon reasonable request, the data are available from the corresponding author pending approval by the Institute of Fundamental Technological Research, Polish Academy of Sciences.

## Abbreviations

The following abbreviations are used in this manuscript:

3D	three dimensional
COV	coefficient of variation
CV	computer-vision-based
DOF	degree of freedom
FE	finite element
LDS	laser displacement sensor
MAC	modal assurance criterion
NSD	normalised standard deviation
ROI	region of interest
SHM	structural health monitoring
ZNCC	zero-normalized cross-correlation function
